# Occupational exposure to asbestos and risk of kidney cancer: an updated meta-analysis

**DOI:** 10.1007/s10654-021-00769-x

**Published:** 2021-06-30

**Authors:** Carlotta Zunarelli, Alessandro Godono, Giovanni Visci, Francesco S. Violante, Paolo Boffetta

**Affiliations:** 1 Department of Medical and Surgical Sciences, University of Bologna, Bologna, Italy; 2 Department of Public Health and Pediatric Sciences, University of Turin, Turin, Italy; 3 Stony Brook Cancer Center, Stony Brook University, Lauterbur Dr, Stony Brook, NY 11794 USA

**Keywords:** Asbestos, Kidney cancer, Occupational exposure, Meta-analysis

## Abstract

Limited information is available on carcinogenicity of asbestos on non-respiratory organs. We aimed at conducted an updated systematic review and meta-analysis of cohort studies on occupational exposure to asbestos and risk of kidney cancer. We searched through three databases, PubMed, Embase and Scopus for article published after 2000, and after eliminating duplicates and non-relevant studies, we identified 13 studies. We combined their results with those of 31 non-overlapping studies included in a previous review up to 2000. We conducted a meta-analysis based on random-effects models. The pooled relative risk of kidney cancer for asbestos exposure was 0.94 (95% confidence interval, 0.84–1.04), with no differences according to type of asbestos fiber, geographic region, period of exposure, or estimated quality of the study. Our results showed a lack of association between occupational asbestos exposure and risk of kidney cancer.

**Supplementary Information:**

The online version contains supplementary material available at 10.1007/s10654-021-00769-x.

## Introduction

In 1973 the International Agency for Research on Cancer (IARC) declared asbestos in all its commercial forms as established human carcinogen [1].

However, worldwide asbestos exposure has not yet stopped. The Global Burden of Disease (GBD) project estimates that 125 million people are exposed to asbestos globally each year [2], with chrysotile accounting for more than 95% of all the asbestos used globally [3].

Asbestos-induced carcinogenesis is a complex event resulting from different factors including physicochemical characteristics of the fibers (dimension, surface reactivity and chemical composition), time and dose of exposure and, finally, host genetic determinants [4].

Occupational and non-occupational asbestos exposures play a well-known role in determining of asbestosis, pulmonary function decline, lung cancer and mesothelioma [5–7]; in addition, several studies investigated relationship between workplace asbestos exposure and others type of cancer.

Although asbestos damages primarily the airways (oral cavity, pharynx, larynx, and lung), some studies suggested a correlation between asbestos occupational exposure and kidney cancer [3].

Kidney cancer is the 14th most common cancer worldwide and ninth most common cancer in Europe. The incidence varies globally, with the highest rates in more developed regions such as North America and Europe and the lowest rates in Asia and Africa [8].

Apart from genetic factors and family history of kidney cancer, established risk factors such as smoking, obesity and hypertension are still prevalent. In addition to such lifestyle factors, environmental agents (such as solvents [9, 10], including in particular trichloroethylene (TCE) [11], pesticides, dusts, diesel and polycyclic aromatic hydrocarbons (PAH) [12]. and medications are thought to be important determinants as well. Physical inactivity, excessive alcohol consumption, unhealthy body weight and poor dietary habits could account for more than 20% of cancer cases [13].

Association between asbestos exposure and kidney cancer remains controversial; to contribute to clarify this issue we conduct an update of previous systematic review and meta-analysis in order to search more recent data about the role of occupational exposure to asbestos fibers in determining kidney cancer.

## Methods

We conducted a systematic review and meta-analysis of cohort studies of workers employed in industries entailing asbestos exposure, according to the PRISMA guidelines [14]. A PRISMA checklist is included as Appendix 1. We retained the studies included in the meta-analysis by Sali and Boffetta [15], and performed a systematic search of literature published from 2001 to May 2020. Three different databases were searched: MEDLINE (PubMed), Scopus and Ovid (Embase). We used a string included all types of cancer and exposure to asbestos (without limitation of types of employment or cancer); the string is reported in Appendix 2.

The study protocol was uploaded on the PROSPERO database and is available from the authors.

### Selection of studies

Searching through databases, 1443 potentially relevant articles were identified. First, two authors (PB and CZ) independently screened papers the titles and abstracts of the articles and selected those matching the inclusion and exclusion criteria. After that, the same two authors read full texts of papers that were considered potentially eligible and selected those that reported relevant results. In case of disagreements, consensus was reached.

We also searched for additional articles through the lists of recent reviews and metanalyses and those of the articles retained for the meta-analysis.

Inclusion criteria of studies for the meta-analysis were (1) Design: cohort and nested case–control study; (2) Asbestos exposure: workers with exclusive or predominant occupational exposure to asbestos (3) Outcome: incidence or mortality due to kidney cancer (C64-ICD10).

Exclusion criteria consisted in (1) Design: community-based studies, cross-sectional and case–control studies (2) type of exposure to asbestos: we excluded studies of workers who might have occasional, low-level exposure to asbestos, as well as exposure to other known carcinogens, such as seamen, bitumen workers, and mechanics.

**Fig. 1:**
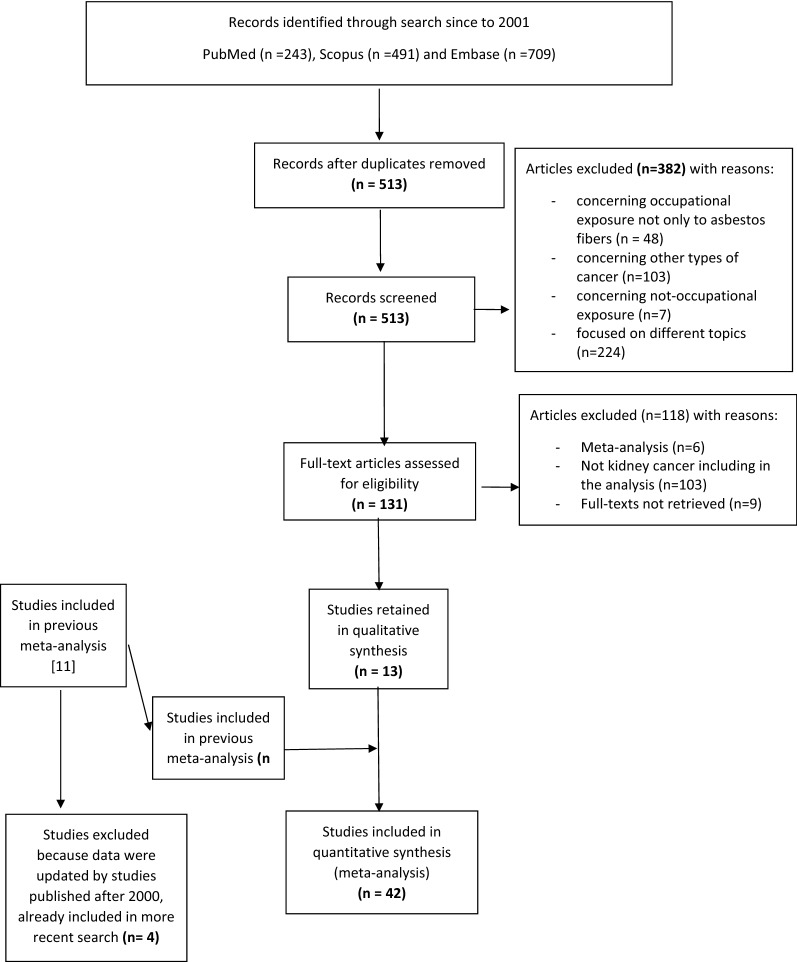
Flowchart for the identification of articles for the meta-analyses

In Fig. 1 is shown the flowchart for selection of the studies in meta-analysis. Details on the studies retained in each step of the process are available from the authors.

### Extraction of data

For each mortality study we extracted the standardized mortality ratio (SMR) of kidney cancer and its 95% confidence interval (CI); when these measures were not directly available from publications, we calculated them. Similarly, we abstracted or calculated (if not specified in the text) the standardized incidence ratio (SIR) corresponding CI for cancer incidence studies. Incidence studies obtained data from cancer registries, while mortality studies were based on information from death certificates.

In addition, we extracted data about (1) type of industry or occupation of employment (for example shipyard, asbestos textile or asbestos mining); (2) asbestos type, classified as pure chrysotile (Ch), predominantly chrysotile (PCh), amphiboles (Am), and mixed or unspecified (Mix); (3) geographic region, categorized as Europe, North America, and others; (4) source of diagnosis: cancer registry, death certificate; (5) composition of the cohort by sex: female when cohort was formed up to 80% by women, male when there were up to 80% of men, and mixed in case of both male and women comprised less than 80% of the entire cohort; (6) years of employment; (7) proportion of cohort members lost to follow-up; (8) period of first exposure: we distinguished cohorts first exposed to asbestos before 1945, from 1946 to 1960 and after 1961; (9) duration of exposure and time since first exposure (latency).

We also performed quality assessment (QA) according to NIH score for quality assessment tool for Observational Cohort and Cross-Sectional Studies. We modified predefined criteria basing on our object of study [16]; we retained thirteen criteria and classified studies into three categories based on the score obtained: (1) high quality (20.25–15 points); (2) medium quality (15–10 points); (3) low quality (0–10 points).

### Statistical analysis

We conducted random-effects [17]. meta-analyses of the RR both combining results for cancer incidence and mortality (when both were available for the same cohort, we used the SIR), and separating them. We also conducted stratified meta-analyses according to the characteristics described above, to explore potential sources of heterogeneity, and a cumulative meta-analysis, according to year of publication of subsequent studies.

To evaluated results stability, we performed sensitivity analyses by quality score and repeated the meta-analysis after excluding one study at a time. Furthermore, we considered the funnel plot and performed the Egger’s regression asymmetry test to assess publication bias [18].

Analyses were performed by STATA16 program [19], using specific commands *metan*, *metabias*, and *metafunnel*.

## Results

In total, 927 publications were identified from search conducted among three databases from 2001 to 2020. We found out 243 results from PubMed (MEDLINE), 491 from Scopus and 193 from Embase, of which 414 were removed because they were duplicates.

We screened the titles and abstracts of 513 articles and rejected 382 of them because not relevant (Fig. 1), and retained 131 articles as potentially eligible. After reading full-texts of 122 of them (full texts were not available for 9), we excluded 109 articles that did not meet inclusion criteria.

We included in the review 13 studies retrieved from the search since 2001, in addition to 29 non-overlapping studies out of 33 included in previous meta-analysis [15]. Out of the 42 studies, we abstracted 54 results from 51 separate cohorts (Table 1).

**Table 1:** Selected characteristics of cohorts included in the meta-analysis

References	Industry	Years of employment	Asbestos type	Country	Occur-rence	Sex	N workers	N	RR (95% CI)
Selikoff et al. 1979a [20]	Insulators	1943	M/U	USA	Mo	M	632	2	1.50 (0.18–5.43)
Selikoff et al. 1979b [21]	Shipyard	1943–1962	M/U	USA	Mo	M	833	1	5.00 (0.13–27.86)
Thomas et al. 1982 ^a^ [22]	Cement	1936–1977	Ch	UK	Mo	M	1592	1	0.5 (0.01–2.79)
Acheson et al. 1982 ^a^ [23]	Gas mask mft	1939–1980	Ch	UK	Mo	F	570	0	0 (0–7.83)
Acheson et al. 1982 ^a^ [23]	Gas mask mft	1939–1980	Am	UK	Mo	F	757	0	0 (0–5.86)
Acheson et al. 1982 ^a^ [23]	Gas mask mft	1947–1979	Am	UK	Mo	M	4820	4	1.17 (0.32–3.00)
Ohlson et al.^a^ [24]	Railroad	1939–1980	M/U	Sweden	Mo	M	3297	10	0.94 (0.45–1.73)
Ohlson & Hogstedt^a^ [25]	Cement	1943–1976	Ch	Sweden	Mo	M	1176	3	0.94 (0.19–2.74)
Peto et al.[26]	Textile	1933–1974	Ch	UK	Mo	M	3211	1	0.25 (0.01–1.37)
Peto et al. 1985 [26]	Textile	1933–1983	Ch	UK	Mo	F	283	0	0 (0–20.49)
Newhouse et al. 1985 ^a^ [27]	Textile, cement, laggers	1933–1980	M/U	UK	Mo	MF	5522	2	0.54 (0.07–1.96)
Kolonel et al. 1985 ^a^ [28]	Shipyard	1940–1993	M/U	USA	Mo	M	7971	9	0.95 (0.43–1.8)
Gardner et al.^a^ [29]	Cement	1930–1983	Ch	UK	Mo	MF	2173	0	0 (0–1.72)
Enterline et al. 1987 [30]	Mix	retired 1941–1980	M/U	USA	Mo	M	1074	7	2.76 (1.11–5.68)
Hughes et al.^a^ [31]	Cement	1940–1982	Ch	UK	Mo	M	6931	7	1.32 (0.53–2.72)
Armstrong et al.^a^ [32]	Miners and millers	1943–1966	Am	Australia	Inc	MF	7328	17	1.03 (0.6–1.65)
Armstrong et al.^a^ [32]^b^	Miners and millers	1943–1966	Am	Australia	Mo	MF	7328	7	0.60 (0.24–1.24)
Szeszenia-Dabrowska et al. 1988 ^a^ [33]	Product mft	1945–1985	Ch	Poland	Mo	F	1190	0	0 (0–7.69)
Ribak et al. 1989 ^a^ [34]	Product mft	1941–1945	Am	USA	Mo	M	820	3	1.76 (0.36–5.16)
Albin et al.^a^ [35]	Cement	1907–1977	Ch	Sweden	Inc	M	2567	7	0.85 (0.36–1.83)
Albin et al. 1990 ^a^ [35]^b^	Cement	1907–1977	Ch	Sweden	Mo	M	2567	10	0.84 (0.40–1.54)
Neuberger & Knudi 1990 ^a^ [36]	Cement	1950–1986	Ch	Austria	Mo	MF	2816	1	0.56 (0.01–3.10)
Selikoff & Seidman[37]	Insulators	< 1953	M/U	USA, Canada	Mo	M	17,800	32	1.70 (1.16–2.39)
Sandén et al.^a^ [38]	Shipyard	1977–1987	Am	Sweden	Inc	M	3893	6	0.48 (0.18–1.05)
Tulchinsky et al.^a^ [39]	Cement	1953–1992	Ch	Israel	Mo	M	3057	5	1.04 (0.34–2.42)
McDonald et al.[40]	MMPM	1976–1988	Ch	Canada	Mo	M	5335	13	1.00 (0.53–1.71)
Meurman et al.^a^ [41]	Miners and millers	1918–1995	Am	Finland	Inc	MF	1045	6	1.85 (0.68–4.03)
Meurman et al.^a^ [41]^b^	Miners and millers	1918–1995	Am	Finland	Mo	MF	1045	1	0.68 (0.02–3.76)
Dement et al.^a^ [42]	Textile	1940–1990	Ch	USA	Mo	MF	3022	4	0.87 (0.24–2.23)
Rösler & Woitowitz^a^ [43]	Mix	1913–1968	M/U	Germany	Mo	MF	3988	3	0.53 (0.11–1.55)
Englund^a^ [44]	Insulators	1967–1991	M/U	Sweden	Inc	M	1690	8	1.40 (0.61–2.77)
Wilczynska et al.^a^ [45]	Product mft	1945–1990	Ch	Poland	Inc	M	2403	2	0.61 (0.07–2.19)
Raffn et al.^a^ [46]	Cement	1928–1984	Am	Denmark	Inc	MF	8580	22	0.92 (0.58–1.40)
Germani et al.^a^ [47]	Workers with asbestosis	NA	M/U	Italy	Mo	M	3417	3	0.73 (0.15–2.14)
Szeszenia-Dabrowska et al.^a^ [48]	Cement	1945–1990	M/U	Poland	Mo	MF	4712	2	0.55 (0.07–2.00)
Puntoni et al.^a^ [49]	Shipyard	NA	M/U	Italy	Mo	M	82	0	0 (0–18.45)
Berry et al.[50]	Mix	NA	M/U	UK	Mo	MF	over 5100	2	0.55 (0.07–1.99)

References	Industry	Years of employment	Asbestos type	Country	Occur-rence	Sex	N workers	N	RR (95% CI)
Ulvestad et al.[51]	Mix	1942–1976	Ch	Norway	Inc	M	541	4	1.3 (0.4–3.4)
Ulvestad et al.[52]	Insulators	1920–1976	M/U	Norway	Inc	M	1116	6	1.00 (0.4–2.2)
Kjærheim et al.[53]	Lighthouse keepers	1917–1967	Ch	Norway	Mo	M	726	8	1.4 (0.6–2.7)
Harding et al.[54]	Mix	NA	M/U	UK	Mo	MF	98,117	114	1.53 (1.26–1.83)
Kimiko et al.[55]	Shipyard (laggers)	1947–2007	M/U	Japan	Mo	M	88	0	0.00 (0.00–23.68)
Kimiko et al.[55]	Shipyard (boiler repair)	1947–2007	M/U	Japan	Mo	M	156	1	3.38 (0.09–18.82)
Wang et al.[56]	Textile	1972–2008	Ch	China	Mo	M	577	1	3.03 (0.53–17.17)
Wang et al.^a^ [56]	Textile	1972–2008	Ch	China	Mo	F	277	0	0.00 (0.00–30.6)
Van den Borre et al.[57]	Mix (MW)	1991	M/U	Belgium	Mo	M	1743	2	1.88 (0.23–6.8)
Van den Borre et al.[57]	Mix (NMW)	1991	M/U	Belgium	Mo	M	313	1	3.24 (0.08–18.06)
Wei-Te et al.[58]	Shipyard	1985–2008	M/U	Taiwan	Inc	M	4427	4	1.28 (0.43–3.85)
Pira et al.[59]	Textile	1946–1984	Ch	Italy	Mo	M	894	1	0.35 (0.01–1.92)
Pira et al.[59]	Textile	1946–1984	Ch	Italy	Mo	F	1083	3	2.46 (0.51–7.18)
Ferrante et al.[60]	Mix	1970–2010	M/U	Italy	Mo	M	46,060	157	0.98 (0.83–1.14)
Ferrante et al.[60]	Mix	1970–2010	M/U	Italy	Mo	F	5741	6	0.59 (0.22–1.29)
Pira et al.[61]	Mining	1930–1989	Ch	Italy	Mo	M	1056	2	0.62 (0.07–2.23)
Barbiero et al.[62]	Shipyard	1974–1994	M/U	Italy	Inc	M	2488	11	0.82 (0.41–1.47)

^a^Results not available from texts, but calculated by us or obtained after contacting the Authors

^b^Excluded from the main meta-analysis

NA, not-available; MW, manual workers; NMW, non-manual workers; MMPM, miners, millers, product mft; Ch, predominantly chrysotile; Am, predominantly amphiboles; M/U, mixed, unknown; Inc, cancer incidence; Mo, mortality; M, predominantly men; W, predominantly women; MW, men and women; N, number of observed events; RR, relative risk (SMR or SIR); CI, confidence interval

Among the 51 cohorts, 34 comprised predominantly men, six predominantly women, and 11 both men and women. Furthermore, 34 cohorts were from Europe, nine from North America and eight from other countries. The main type of asbestos fiber was chrysotile in 21 cohorts and amphiboles in seven, while in the remaining 23 cohorts it was not possible to distinguish between the two types. Results on kidney cancer mortality were available for 43 cohorts, and those on cancer incidence for 11 cohorts (for three cohorts both types of results were available).

**Fig. 2:**
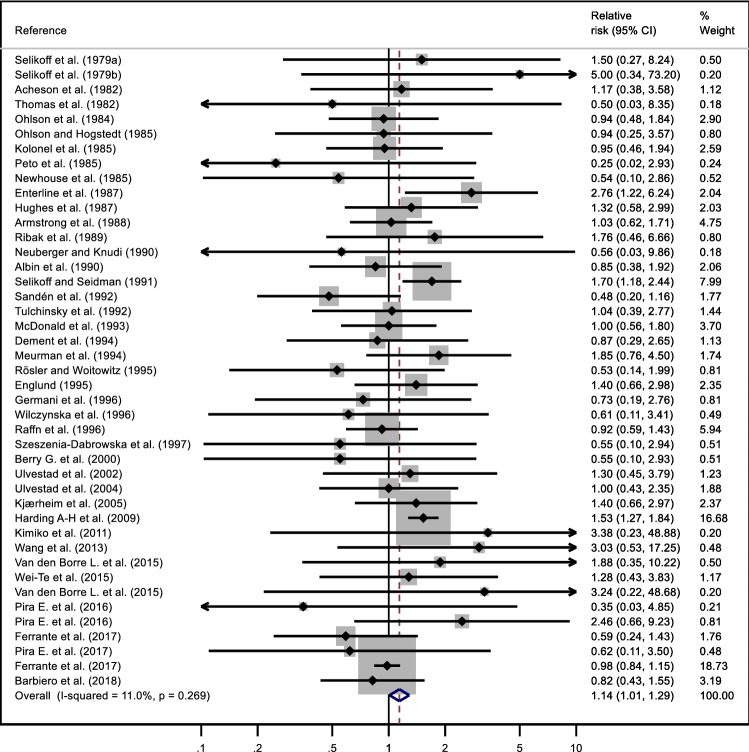
Forest plot of meta-analysis of results on risk of kidney cancer among occupational cohorts exposed to asbestos

Figure 2 presents the pooled results based on random-effected model. The pooled RR was 1.14 (95% CI 1.04–1.29); eight results were excluded from the meta-analysis because they were based on zero observed events There was limited evidence of heterogeneity between studies (*p* = 0.3; I2 = 11%). The summary RR of results on cancer incidence was 0.98 (95% CI 0.79–1.22) with no evidence of heterogeneity between studies (*p* = 0.8, I^2^ = 0%); that of results on mortality 1.15 (95% CI 0.99–1.34; *p* = 0.2; I2 = 19%;).

Table 2 shows the results of stratified analyses, that revealed no difference by asbestos type, region, sex, or period of first exposure.

**Table 2:** Results of meta-analyses stratified according to selected characteristics

Characteristic		N risk estimates^c^	RR (95% CI)	p-het
Type of asbestos fiber	Amosite	6	1.00 (0.74–1.35)	0.43
	Chrysotile	16	1.07 (0.81–1.41)	
	Unspecified	21	1.17 (0.96–1.43)	
Geographic region	North America	9	1.45 (1.14–1.85)	0.13
	Europe	29	1.05 (0.90–1.22)	
	Others	5	1.15 (0.77–1.72)	
Gender	Men	31	1.08 (0.96–1.21)	0.10
	Women	2	1.10 (0.27–4.42)	
	Mixed	10	1.12 (0.85–1.47)	
Year of first employment	< 1945	21	1.09 (0.90–1.32)	0.001
	1945–1960	9	1.47 (1.09–1.99)	
	> 1960	10	0.97 (0.85–1.12)	
	Not specified	3	1.27 (0.76–2.13)	

RR, meta-relative risk, based on random-effects model; CI, confidence interval; p-het, p-value of test for heterogeneity of strata-specific RR

^c^Cohort-specific results based on 0 observed events are excluded from the meta-analys

We performed quality assessment by NIH score for studies included in our analysis published after 2000. Only two studies were considered good quality because of good exposure assessment (several measurements), low proportion of lost to follow up and analysis adjusted for others risk factors of kidney cancer. Eight studies were of moderate quality, only few of them included a satisfactory description of sample size justification or prior measurement of the outcome.

Some authors considered also others circumstances of asbestos exposure. Kjaerheim et al. (2005) studied the effect of exposure to asbestos in drinking water; the SIR for kidney cancer was 1.4 (95% CI 0.6–2.7). Van den Borre et al. (2015) distinguished between manual workers and non-manual workers exposed to asbestos, and found no differences between the two groups, with SMR equal to 1.88 (95% CI 0.23–6.80) and 3.24 (95% CI 0.08–18.06), respectively.

Only two studies reported data about latency or duration of exposure [33, 34].

**Fig. 3:**
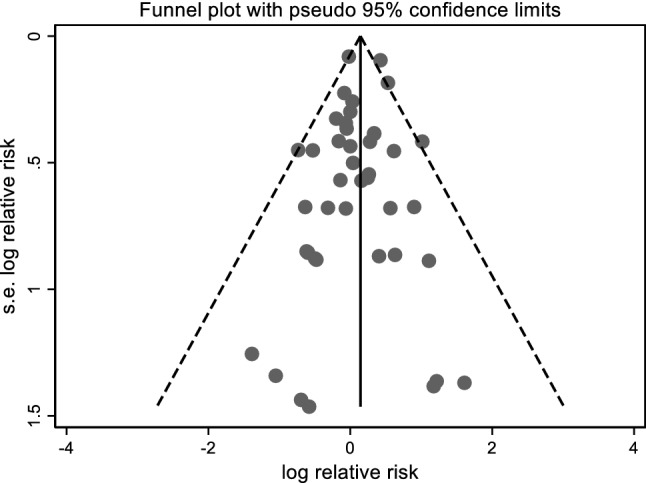
Funnel plot of Egger’s test to assess publication bias among studies included in the meta-analysis

As shown in Fig. 3, we found no evidence of publication bias in the main results of the cohorts included in the meta-analysis (p-value of Egger’s test, 0.4 for mortality studies and 0.9 for incidence studies).

## Discussion

Kidney cancer is the 14th common cancer in the world and, while 50% of kidney cancer are not attributable to known risk factors for that malignancy [8], only few studies considered its association with asbestos exposure. Furthermore, is difficult to observed cohorts of workers exposed only to asbestos fibers, as many of them, entail exposure to other potential kidney cancerogenic risk factors.

Our results show a lack of association between occupational asbestos exposure and kidney cancer; indeed, pooled analysis shows relative risk 1.1 (95% CI 1.0–1.3). This result was confirmed in subgroup analyses stratified by type of asbestos fibers, country, sex or period of first exposure. These results are also consistent with those of a previous meta-analysis of studies published up to 2000 [15].

Our study suffers from several limitations. The most important problem is that seven studies we selected after systematic review of literature showed no cases of kidney cancers among cohorts exposed to asbestos, and were excluded from the meta-analysis, that was based on log-transformed measures of associations. In order to assess the magnitude of the bias away from the null, we performed also a meta-analysis without logarithm transformation both including and excluding studies with no observed cases or deaths, and the results were not materially changed by the exclusion of these latter studies.

Our analysis included a small number of studies based on cancer incidence, and results of studies on cancer mortality might be affected by determinants of survival, such as access to effective treatment. Furthermore, many studies evidenced low accuracy of COD, especially among elderly people who more frequently have a multiple comorbidity before death (62).

The incidence of kidney cancer is often underestimated as it is a tumor that is diagnosed late, sometimes when metastases are already present in other sites and it is not always assumed that it is possible to trace the primary tumor (especially as regards data relating to tumors that have arisen several years ago, when the analysis of the tumor histotype was not so frequent and therefore it was even more difficult to trace the starting site of the tumor).

Furthermore, only three studies [51, 54, 63] considered tobacco smoking as potential confounding factor, and none included obesity, alcohol consumption, diabetes, hypertension, analgesic therapy in the analysis [64].

In addition only two papers (mentioned before) reported data about latency and duration of exposure, for that reason we decided to not perform a dose–response meta-analysis.

Finally, exposure assessment remains an issue in occupation cohort studies based on job titles or industries of employment, without direct exposure measurements. Such approach does not distinguish between circumstances at different levels of exposure, leading to possible underestimate of the effects.

We decided to review the data about risk for kidney cancer among workers exposed to asbestos due to well-known role of asbestos fibers in determining carcinogenic process in other organs and some supporting biological data. Boor et al. showed an increase of renal fibrosis and production of inflammatory mediators locally in rats exposed to amiosite fibers [65]. and that kind of data support the fact that asbestos fibers could be transported by blood to renal cells and there could trigger inflammatory process and, subsequently, genetic alteration.

More evidence of capacity for asbestos fibers to reach kidney has been provided by necropsy studies of humans; moreover, asbestos fibers have been founded in human urine [66].

A strength of our study is the large number of cohort studies included in the meta-analysis. Furthermore, the limited heterogeneity between study results, the consistency of results across different subgroups of studies, and the lack of evidence of publication bias support the main conclusion of our meta-analysis.

In conclusion our updated meta-analysis provided additional evidence to the hypothesis of a lack of association between occupational exposure to asbestos and risk of kidney cancer.

## Supplementary Information

Below is the link to the electronic supplementary material.

Supplementary file1 (DOCX 18 KB)

## Data Availability

All the primary data are available from the first Author.

## Code availability

All the software code are available from the first Author.

## References

[1] International Agency of Research on Cancer (2012). Arsenic, metals, fibers, and dusts. IARC Monogr Eval Carcinog Risk Hum.

[2] Concha-Barrientos M. et al. Comparative Quantification of Health Risks:Global and Regional Burden of Diseases Attributable of Selected Major Risk Factors. Geneva, Switzerland: World Health Organization;2004:1651e801

[3] LaDou J (2004). The asbestos cancer epidemic. Environ Health Perspect.

[4] Padmore (2017). Quantitative analysis of the role of fiber length on phagocytosis and inflammatory response by alveolar macrophages. Biochim Biophys Acta Gen Subj.

[5] Lacourt A (2013). Pleural mesothelioma and occupational coexposure to asbestos, mineral wool, and silica. Am J Respir Crit Care Med.

[6] Markowitz SB (2013). Asbestos, asbestosis, smoking, and lung cancer: new findings from the North American insulator cohort. Am J Respir Crit Care Med.

[7] Rödelsperger K (2001). Asbestos and man-made vitreous fibers as risk factors for diffuse malignant mesothelioma: results from a German hospital-based case-control study. Am J Ind Med.

[8] Bray F, Ferlay J, Soerjomataram I, Siegel RL, Torre LA, Jemal A (2018). Global cancer statistics 2018: GLOBOCAN estimates of incidence and mortality worldwide for 36 cancers in 185 countries. CA Cancer J Clin.

[9] American Cancer Society (2016). Cancer Facts & Figures 2016.

[10] Lohi J, Kyyrönen P, Kauppinen T, Kujala V, Pukkala E (2008). Occupational exposure to solvents and gasoline and risk of cancers in the urinary tract among Finnish workers. Am J Ind Med.

[11] Schlehofer B, Heuer C, Blettner M, Niehoff D, Wahrendorf J (1995). Occupation, smoking and demographic factors, and renal cell carcinoma in Germany. Int J Epidemiol.

[12] Scott CS, Jinot J (2011). Trichloroethylene and cancer: systematic and quantitative review of epidemiologic evidence for identifying hazards. Int J Environ Res Public Health.

[13] Mandel JS, McLaughlin JK, Schlehofer B, Mellemgaard A, Helmert U, Lindblad P, McCredie M, Adami HO (1995). International renal-cell cancer study. IV Occupation Int J Cancer.

[14] Moher D, Liberati A, Tetzlaff J, Altman DG (2009). PRISMA Group. Preferred reporting items for systematic reviews and meta-analyses: the PRISMA statement. Ann Int Med..

[15] Sali D, Boffetta P (2000). Kidney cancer and occupational exposure to asbestos: a meta-analysis of occupational cohort studies. Cancer Causes Control.

[16] https://www.nhlbi.nih.gov/health-topics/study-quality-assessment-tools. Accessed on 10.6.2020

[17] DerSimonian R, Laird N (1986). Meta-analysis in clinical trials. Control Clin Trials.

[18] Egger M, Davey Smith G, Schneider M, Minder C (1997). Bias in meta-analysis detected by a simple, graphical test. BMJ.

[19] StataCorp.  (2019). Stata Statistical Software: Release 16.

[20] Selikoff IJ, Hammond EC, Seidman H (1979). Mortality experience of insulation workers in the United States and Canada, 1943–1976. Ann N Y Acad Sci.

[21] Thomas HF, Benjamin IT, Elwood PC, Sweetnam PM (1982). Further follow-up study of workers from an asbestos cement factory. Br J Ind Med.

[22] Acheson ED, Gardner MJ, Pippard EC, Grime LP (1982). Mortality of two groups of women who manufactured gas masks from chrysotile and crocidolite asbestos: a 40-year follow-up. Br J Ind Med.

[23] Ohlson CG, Klaesson B, Hogstedt C (1984). Mortality among asbestos-exposed workers in a railroad workshop. Scand J Work Environ Health.

[24] Ohlson CG, Hogstedt C (1985). Lung cancer among asbestos cement workers A Swedish cohort study and a review. Br J Ind Med.

[25] Peto J, Doll R, Hermon C, Binns W, Clayton R, Goffe T (1985). Relationship of mortality to measures of environmental asbestos pollution in an asbestos textile factory. Ann Occup Hyg.

[26] Kolonel LN, Yoshizawa CN, Hirohata T, Myers BC (1985). Cancer occurrence in shipyard workers exposed to asbestos in Hawaii. Cancer Res.

[27] Gardner MJ, Winter PD, Pannett B, Powell CA (1986). Follow up study of workers manufacturing chrysotile asbestos cement products. Br J Ind Med.

[28] Enterline PE, Hartley J, Henderson V (1987). Asbestos and cancer: a cohort followed up to death. Br J Ind Med.

[29] Hughes JM, Weill H, Hammad YY (1987). Mortality of workers employed in two asbestos cement manufacturing plants. Br J Ind Med.

[30] Armstrong BK, de Klerk NH, Musk AW, Hobbs MS (1988). Mortality in miners and millers of crocidolite in Western Australia. Br J Ind Med.

[31] Szeszenia-Dabrowska N, Wilczyńska U, Szymczak W (1988). Mortality among female workers in an asbestos factory in Poland. Pol J Occup Med.

[32] Ribak J, Seidman H, Selikoff IJ (1989). Amosite mesothelioma in a cohort of asbestos workers. Scand J Work Environ Health.

[33] Albin M, Jakobsson K, Attewell R, Johansson L, Welinder H (1990). Mortality and cancer morbidity in cohorts of asbestos cement workers and referents. Br J Ind Med.

[34] Neuberger M, Kundi M (1990). Individual asbestos exposure: smoking and mortality–a cohort study in the asbestos cement industry. Br J Ind Med.

[35] Selikoff IJ, Seidman H (1991). Asbestos-associated deaths among insulation workers in the United States and Canada, 1967–1987. Ann N Y Acad Sci.

[36] Sandén A, Järvholm B, Larsson S, Thiringer G (1992). The risk of lung cancer and mesothelioma after cessation of asbestos exposure: a prospective cohort study of shipyard workers. Eur Respir J.

[37] Tulchinsky TH, Ginsberg GM, Shihab S, Goldberg E, Laster R (1992). Mesothelioma mortality among former asbestos-cement workers in Israel, 1953–90. Isr J Med Sci.

[38] McDonald JC, Liddell FD, Dufresne A, McDonald AD (1993). The 1891–1920 birth cohort of Quebec chrysotile miners and millers: mortality 1976–88. Br J Ind Med.

[39] Meurman LO, Pukkala E, Hakama M (1994). Incidence of cancer among anthophyllite asbestos miners in Finland. Occup Environ Med.

[40] Dement JM, Brown DP, Okun A (1994). Follow-up study of chrysotile asbestos textile workers: cohort mortality and case-control analyses. Am J Ind Med.

[41] Rösler JA, Woitowitz HJ (1995). Recent data on cancer due to asbestos in Germany. Med Lav.

[42] Englund A (1995). Recent data on cancer due to asbestos in Sweden. Med Lav.

[43] Wilczyńska U, Szeszenia-Dabrowska N, Szymczak W (1996). Umieralność z powodu nowotworów złośliwych wśród mezczyzn zawodowo narazonych na pył azbestu [Mortality from malignant neoplasms in men occupationally exposed to asbestos dust].. Med Pr.

[44] Raffn E, Villadsen E, Engholm G, Lynge E (1996). Lung cancer in asbestos cement workers in Denmark. Occup Environ Med.

[45] Germani D, Grignoli M, Belli S, Bruno C, Maiozzi P, Anibaldi L, Raparelli O, Comba P (1996). Studio di mortalità dei titolari di rendita per asbestosi in Italia (1980–1990) [A mortality study of recipients of compensation for asbestosis in Italy (1980–1990)].. Med Lav.

[46] Szeszenia-Dabrowska N, Wilczyńska U, Szymczak W (1997). Ryzyko choroby nowotworowej w przemyśle azbestowo-cementowym w Polsce [Cancer risk in asbestos-cement industry workers in Poland].. Med Pr.

[47] Berry G, Newhouse ML, Wagner JC (2000). Mortality from all cancers of asbestos factory workers in east London 1933–80. Occup Environ Med.

[48] Ulvestad B, Kjaerheim K, Martinsen JI, Damberg G, Wannag A, Mowe G, Andersen A (2002). Cancer incidence among workers in the asbestos-cement producing industry in Norway. Scand J Work Environ Health.

[49] Ulvestad B, Kjaerheim K, Martinsen JI, Mowe G, Andersen A (2004). Cancer incidence among members of the Norwegian trade union of insulation workers. J Occup Environ Med.

[50] Kjaerheim K, Ulvestad B, Martinsen JI, Andersen A (2005). Cancer of the gastrointestinal tract and exposure to asbestos in drinking water among lighthouse keepers (Norway). Cancer Causes Control.

[51] Harding AH, Darnton A, Wegerdt J, McElvenny D (2009). Mortality among British asbestos workers undergoing regular medical examinations (1971–2005). Occup Environ Med.

[52] Tomioka K, Natori Y, Kumagai S, Kurumatani N (2011). An updated historical cohort mortality study of workers exposed to asbestos in a refitting shipyard, 1947–2007. Int Arch Occup Environ Health.

[53] Wang X, Lin S, Yu I, Qiu H, Lan Y, Yano E (2013). Cause-specific mortality in a Chinese chrysotile textile worker cohort. Cancer Sci.

[54] Van den Borre L, Deboosere P (2015). Enduring health effects of asbestos use in Belgian industries: a record-linked cohort study of cause-specific mortality (2001–2009). BMJ Open.

[55] Wu WT, Lin YJ, Li CY, Tsai PJ, Yang CY, Liou SH, Wu TN (2015). Cancer attributable to asbestos exposure in shipbreaking workers: a matched-cohort study. PLoS ONE.

[56] Pira E, Romano C, Violante FS, Farioli A, Spatari G, La Vecchia C, Boffetta P (2016). Updated mortality study of a cohort of asbestos textile workers. Cancer Med.

[57] Ferrante D, Chellini E, Merler E, Pavone V, Silvestri S, Miligi L, Gorini G, Bressan V, Girardi P, Ancona L, Romeo E, Luberto F, Sala O, Scarnato C, Menegozzo S, Oddone E, Tunesi S, Perticaroli P, Pettinari A, Cuccaro F, Mattioli S, Baldassarre A, Barone-Adesi F, Cena T, Legittimo P, Marinaccio A, Mirabelli D, Musti M, Pirastu R, Ranucci A, Magnani C (2017). the working group. Italian pool of asbestos workers cohorts: mortality trends of asbestos-related neoplasms after long time since first exposure. Occup Environ Med.

[58] Pira E, Romano C, Donato F, Pelucchi C, Vecchia C, Boffetta P (2017). Mortality from cancer and other causes among Italian chrysotile asbestos miners. Occup Environ Med.

[59] Barbiero F, Zanin T, Pisa FE, Casetta A, Rosolen V, Giangreco M, Negro C, Bovenzi M, Barbone F (2018). Cancer incidence in a cohort of asbestos-exposed workers undergoing health surveillance. Int Arch Occup Environ Health.

[60] Kuchiba A, Ishikawa S, Sawabe M (2016). Accuracy of death certificates and assessment of factors for misclassification of underlying cause of death. J Epidemiol.

[61] Chow WH, Dong LM, Devesa SS (2010). Epidemiology and risk factors for kidney cancer. Nat Rev Urol.

[62] Boor P, Casper S, Celec P, Hurbánková M, Beno M, Heidland A, Amann K, Sebeková K (2009). Renal, vascular and cardiac fibrosis in rats exposed to passive smoking and industrial dust fibre amosite. J Cell Mol Med.

[63] Smith AH, Shearn VI, Wood R (1989). Asbestos and kidney cancer: the evidence supports a causal association. Am J Ind Med.

[64] Chow WH, Dong LM, Devesa SS (2010). Epidemiology and risk factors for kidney cancer. Nat Rev Urol..

[65] Boor P, Casper S, Celec P, Hurbánková M, Beno M, Heidland A, Amann K, Sebeková K (2009). Renal, vascular and cardiac fibrosis in rats exposed to passive smoking and industrial dust fibre amosite. J Cell Mol Med..

[66] Zaina S, Mastrangelo G, Ballarin MN, Scoizzato L, Carradori G, Fedeli U, Capella S, Belluso E (2016). Urinary asbestos fibers and inorganic particles in past asbestos workers. Arch Environ Occup Health..

